# Supplementary material for: Computational prediction shines light on type III secretion origins
Source: Sci Rep. 2016 Oct 7;6:34516. doi: 10.1038/srep34516 (PMC5054392; doi:10.1038/srep34516)
Supplement: Supplementary Information [file srep34516-s1.doc]

Supplementary Information
for:
Computational prediction shines light on type III secretion origins

Tatyana Goldberg, Burkhard Rost and Yana Bromberg

# Table of Contents for Supplementary Information

S1 Table: Method performance comparison on independent test sets 2

S1 Text: Performance evaluation 3

S2 Text: State-of-the-art predictors for type III effector proteins 4

S1 Figure: Distribution of a typical translated read length 5

S2 Table: Effector predictions from protein fragments 6

S2 Figure: Reliable predictions are more accurate 7

S3 Figure: Reliability of PSI-BLAST and *de novo* predictions 8

S3 Table: Predictions of type III secretion system in 862 bacterial and 90

archaeal proteomes 9

S4 Table: Top 10 most frequent UniProt keywords of bacterial and archaeal

proteins predicted as type III effectors by pEffect 10

S5 Table: Clusters of T3 Orthologs in 36 bacterial genera as annotated in the

T3DB database 11

S6 Table: Experimental evidence for the type III machinery in 10 completely

sequenced bacteria 12

S4 Figure: Type III effector proteins most common in mammalian pathogens 13

S7 Table: Method performance comparison on a fully independent test set 14

# Material

*S1 Table: Method performance comparison on independent test sets*

|  | *UniProt’15_HVAL0_^3^* | | | *UniProt’15_Full_^4^* | | | *T3DB_HVAL0_^5^* | | | *T3DB_Full_^6^* | | |
| --- | --- | --- | --- | --- | --- | --- | --- | --- | --- | --- | --- | --- |
|  | *Acc^7^* | *Cov^7^* | *F_1_^7^* | *Acc^7^* | *Cov^7^* | *F_1_^7^* | *Acc^7^* | *Cov^7^* | *F_1_^7^* | *Acc^7^* | *Cov^7^* | *F_1_^7^* |
| *BPBAac^1^* | 39±20 | 25±15 | 0.31±0.10 | 38±9 | 14±4 | 0.21±0.03 | **83±16** | 44±13 | 0.57±0.13 | **82±8** | 52±8 | 0.64±0.08 |
| *EffectiveT3^1^* | 17±7 | 51±17 | 0.25±0.06 | 31±4 | 38±5 | 0.34±0.03 | 54±14 | 58±15 | 0.56±0.12 | 46±7 | 67±8 | 0.55±0.06 |
| *T3_MM^1^* | - | - | - | 31±5 | 40±6 | 0.35±0.03 | 47±13 | 64±14 | 0.54±0.10 | 50±6 | 73±7 | 0.60±0.06 |
| *Modlab^1^* | 22±8 | 63±15 | 0.32±0.08 | 44±5 | 48±6 | 0.46±0.04 | 64±14 | 62±14 | 0.63±0.14 | 56±7 | 70±7 | 0.62±0.06 |
| *BEAN 2.0^1^* | 19±6 | 73±15 | 0.31±0.07 | 49±4 | 73±5 | 0.58±0.04 | 63±14 | **76±12** | **0.68±0.13** | 62±7 | **86±6** | **0.72±0.07** |
| *De novo^2^* | 90±10* | 69±15 | 0.78±0.16 | **94±3** | 79±5 | 0.86±0.04 | 71±16 | 48±15 | 0.58± 0.13 | 65±8 | 66±8 | 0.65±0.07 |
| *PSI-BLAST^2^* | **98±5*** | 82±12 | **0.89±0.12*** | 91±3 | 95±2 | **0.93±0.04** | 78±17 | 47±14 | 0.58±0.15 | 69±8 | 64±8 | 0.66±0.07 |
| *pEffect^2^* | 90±10* | **86±11** | **0.88±0.12*** | 88±3 | **98±1** | **0.93±0.03** | 72±14 | 58±14 | 0.64± 0.14 | 61±7 | 77±7 | 0.68±0.07 |

^1^BPBAac, EffectiveT3, T3_MM, Modlab and BEAN2.0 as in SupplementaryS2 Text.

^2^*De novo*, PSI-BLAST and pEffect, as in Table 1.

^3^As in Methods, 51 type III effectors extracted from UniProt[^1^](#_ENREF_1) after the 2014_02 release and 691 non-effector bacterial and eukaryotic proteins extracted from Swiss-Prot[^2^](#_ENREF_2) after the same release. Data sets are sequence homology reduced at HVAL<0.

^4^As in Methods, 498 effectors extracted from UniProt after the 2014_08 release and 1,509 non-effector bacterial and eukaryotic proteins extracted from Swiss-Prot after the same release. Data sets are NOT homology reduced.

^5^As in Methods, 66 effectors and 128 non-effector bacterial proteins extracted from the T3DB[^5^](#_ENREF_5) database, sequence homology reduced at HVAL<0.

^6^As in Methods, 218 effectors and 831 non-effector bacterial proteins extracted from the T3DB database, NOT homology reduced.

^7^Acc, Cov, F_1_, as in Supplementary S1 Text. Highest value in each column is in bold.

Note: T3_MM was not able to produce results for the UniProt’15_HVAL0_ data set during manuscript preparation.

** = unrealistic upper bound given by the standard error due to the small data set size.

**S1 Text: Performance evaluation**

We measured accuracy/precision (Eqn. 1) and coverage/recall (Eqn. 2) of all prediction methods using ratios of TP (true positives, *i.e.* correctly predicted effector proteins), FP (false positives, *i.e.* non-effector proteins predicted to be effectors), FN (false negatives, *i.e.* effectors predicted to be non-effector proteins), and TN (true negatives, *i.e.* correctly predicted non-effector proteins).

Accuracy/Precision= (Eqn. 1)

Coverage/Recall= (Eqn. 2)

We combined these two measures into a single F measure value:

F_1_= (Eqn. 3)

Standard error was estimated over 1000 bootstrap test sets. Bootstrapping[^6^](#_ENREF_6) was done by randomly selecting (with replacement) sets of *15%* proteins from the original data set. For each bootstrapped set *i*, the performance (*e.g.* accuracy) *x_i_* was estimated. These 1000 estimates provide standard deviation through their difference from the overall performance *<x>* and the standard error *(*Eqn. 4):

Standard Deviation= (Eqn. 4)

Standard Error=

**S2 Text: State-of-the-art predictors for type III effector proteins**

We used the following state-of-the-art methods that predict bacterial type III effector proteins and are publicly accessible:

1. BPBAac[^7^](#_ENREF_7) uses a Support Vector Machine[^8^](#_ENREF_8) (SVM) to predict type III effectors. Predictions are based on the position-specific amino acid composition (Aac) profiles within 100 N-terminal residues of a protein sequence. BPBAac was trained on non-redundant sets of 154 type III effectors curated manually from literature and 308 non-effectors randomly selected from various bacteria, followed by removal of the known effectors and their homologs. We used BPBAac with its default threshold of 0.50. BPBAac is available at <http://biocomputer.bio.cuhk.edu.hk/softwares/BPBAac>.
2. EffectiveT3[^9^](#_ENREF_9) applies a Naïve Bayesian classifier to predict type III effectors on the basis of various features of the 25 N-terminal residues, including frequencies of amino acids, short peptides, and residues with certain physico-chemical properties. Effective T3 was trained on a positive set of 100 manually curated type III effectors from literature. The negative set comprised 200 non-effector proteins collected by randomly choosing proteins from animal and plant pathogens, omitting known effectors. The method was updated in 2015 through assembling additional 504 verified secreted proteins from T3SEdb. Note that none of these secreted proteins was part pf pEffect’s development set. We used the updated version of the method with its default parameter (minimal score=0.9999) and selected both low and high confidence predictions for our evaluation, as, in our hands, their combination provided best performance results for EffectiveT3. The method is available at: <http://www.effectors.org/>.
3. Modlab[^10^](#_ENREF_10) is another method that employs information from the N-terminal region of an amino acid sequence for effector protein prediction. The consistently stable and through a web server accessible version of Modlab is based on a Neural Network classifier that uses composition of 25 consecutive amino acid residues of the 30N-terminal region of a protein in a sliding window approach. Modlab was developed on a dataset of 575 type III effector proteins (extracted from Swiss-Prot and *Pseudomonas syringae* Hop databases, as well as from literature) and 685 bacterial secreted non-effectors (SignalP and SecretomeP training sets). The maximum pairwise sequence identity in the training data set was 90%. We used the method with its default parameters (N-terminal sequence region of 30 amino acids and Neural Network threshold of 0.4). Modlab is available at <http://gecco.org.chemie.uni-frankfurt.de/T3SS_prediction/T3SS_prediction.html>.
4. T3_MM[^11^](#_ENREF_11) is based on BPBAac and uses Aac profiles of adjacent residues to predict type III effectors. It employs a Markov model to calculate the Aac probability difference between type III effector and non-effector proteins. T3_MM was trained on BPBAac training data. Predictions are made using 100 N-terminal residues. We used T3_MM with its default parameters. T3_MM is available at <http://biocomputer.bio.cuhk.edu.hk/T3DB/T3_MM.php>.
5. BEAN 2.0[^12^](#_ENREF_12) consists of three components that are employed consequently if the preceding one does not predict a protein to be a type III effector. The first component uses BLAST[^13^](#_ENREF_13) to search in a dataset of known type III effector proteins. The second component identifies type III effector proteins-specific PFAM[^14^](#_ENREF_14) domains. Finally, the third component employs an SVM that makes its predictions from an evolutionary profile constructed with HHblits[^15^](#_ENREF_15). Specifically, it uses 50N- and 50 C-terminal amino acids as well as 50-120 amino acids from the intermediate region of a profile. BEAN 2.0 was developed on at 40% sequence identity reduced data set of 243 effector and 486 non-effector proteins extracted from UniProt version 2014_01. We used BEAN 2.0 with its default parameters. BEAN 2.0 is available at <http://systbio.cau.edu.cn/bean/index.php>.

### S1 Fig:


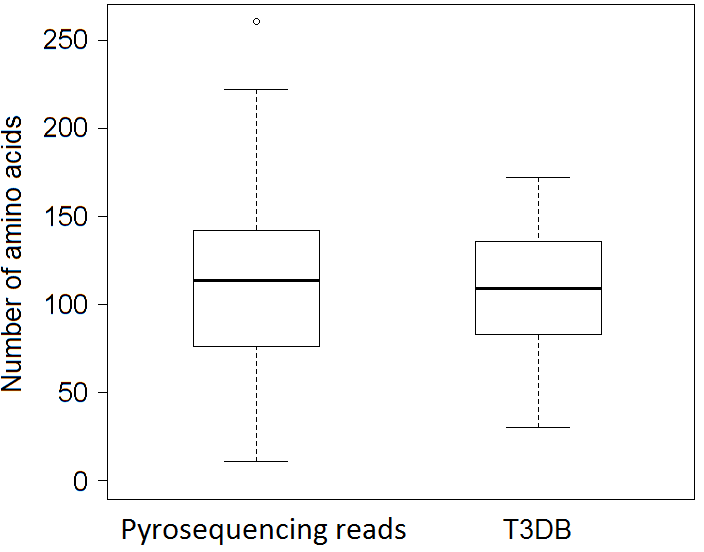


**S1 Fig: Distribution of a typical translated read length.** ”Pyrosequencing reads”: amino acid lengths of open reading frames translated (between start and stop codons) from eight different snow and soil collected metagenomes (collaborator data) using getorf[^16^](#_ENREF_16). “T3DB”: amino acid lengths of randomly picked fragments (one fragment per sequence) from the T3DB_Full_ set (Methods). The distribution of translated read lengths in the T3DB set follows the distribution of read lengths in “real” metagenomic samples.

S2 Table: Effector predictions from protein fragments

| *T3DB_HVAL0_^1^* | *30N Cleaved^2^* | | | *30C Cleaved^3^* | | | *1/3 Randomly Cleaved^4^* | | | *Random Fragments^5^* | | |
| --- | --- | --- | --- | --- | --- | --- | --- | --- | --- | --- | --- | --- |
|  | *Acc* | *Cov* | *F_1_* | *Acc* | *Cov* | *F_1_* | *Acc* | *Cov* | *F_1_* | *Acc* | *Cov* | *F_1_* |
| *BPBAcc* | 67±48 | 6±7 | 0.11±0.07 | 82±16 | 41±14 | 0.55±0.13 | 100±0* | 11±9 | 0.19±0.09 | 50±0 | 2±3 | 0.03±0.04 |
| *EffectiveT3* | 51±19 | 29±13 | 0.37±0.10 | 54±14 | 58±15 | 0.56±0.12 | 60±3 | 58±15 | **0.59±0.12** | 39±17 | 29±13 | 0.33±0.10 |
| *T3_MM* | 45±13 | 53±15 | 0.49±0.11 | 49±13 | 65±14 | 0.56±0.11 | 43±13 | 55±14 | 0.48±0.11 | 43±15 | 45±14 | 0.44±0.10 |
| *Modlab* | 57±25 | 18±11 | 0.28±0.11 | 64±14 | 62±15 | 0.63±0.13 | 54±19 | 39±15 | 0.46±0.12 | 58±22 | 23±13 | 0.33±0.10 |
| *BEAN 2.0* | 67±12 | **71±14** | **0.69±0.13** | 68±14 | **71±13** | **0.70±0.14** | 52±13 | **64±14** | 0.57±0.11 | 74±17 | 42±15 | 0.54±0.14 |
| *De novo* | 70±17 | 42±14 | 0.53±0.12 | 67±18 | 44±14 | 0.53±0.13 | 63±20 | 33±15 | 0.44±0.12 | 70±25 | 21±12 | 0.33±0.11 |
| *PSI-BLAST* | **79±15** | 47±14 | 0.59±0.13 | **77±16** | 45±15 | 0.57±0.14 | **89±15** | 36±15 | 0.52±0.14 | **83±15** | 44±15 | 0.57±0.15 |
| *pEffect* | 70±16 | 56±14 | 0.62±0.12 | 69±15 | 56±14 | 0.62±0.14 | 67±16 | 45±14 | 0.54±0.12 | 74±17 | **48±15** | **0.59±0.14** |

^1^T3DB_HVAL0_ protein set used to produce fragments, as in Methods, 66 effectors and 128 non-effector bacterial sequences. Methods and performance measures as in Supplementary S1 Table. Highest value in each column is in bold.

^2^Approch i: 30 N-terminal amino acids cleaved off.

^3^Approch ii: 30 C-terminal amino acids cleaved off.

^4^Approch iii: Randomly selected two thirds of the protein sequence.

^5^Approch iv: Randomly selected sequence fragments of typical translated read length (average 110 amino acids, Supplementary Fig. S1).

* = unrealistic performance estimate due to the small number of positive predictions (seven in total, of which none are false positives).

### S2 Fig:


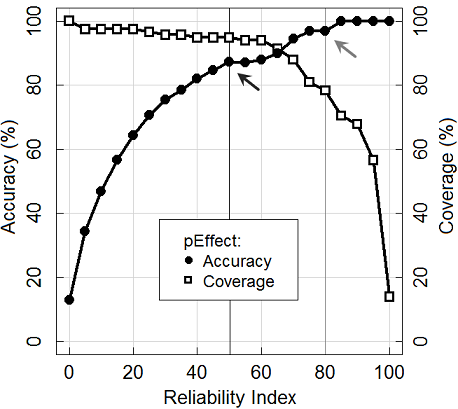


**S2 Fig: Reliable predictions are more accurate.** The figure shows the cumulative percent of accuracy/coverage (Supplementary S1 Text) of pEffect predictions at or above a given reliability index (RI). The graphs were obtained using the homology-reduced *Development set* of 115 type III effector and 3,460 non-effector proteins in five-fold cross-validation. At the reliability score of RI = 50 (black vertical line), 95% of type III effectors are identified at 87% accuracy (black arrow). At a higher reliability score of RI = 80 (gray vertical line), prediction accuracy increases to 97% at the cost of lower coverage of 78% (gray arrow).

### S3 Fig:


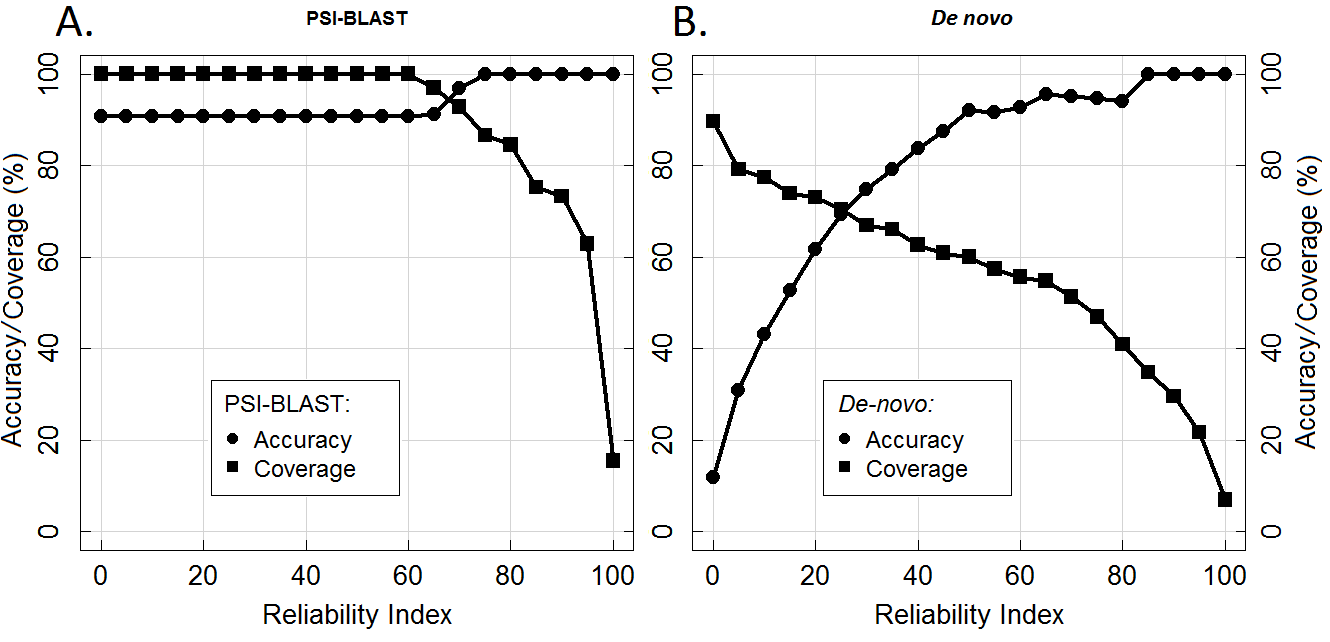


**S3 Fig: Reliability of PSI-BLAST and *de novo* predictions.** The figure shows the cumulative percent of accuracy/coverage (Online Methods) of individual components of pEffect – homology-based PSI-BLAST (A) and *de novo* predictions using SVM (B) – at or above a given reliability index (RI). The graphs were obtained using the homology-reduced cross-validated Development set of 115 type III effector and 3460 non-effector proteins. RIs for *de novo* predictions are read off directly from the SVM output. pEffect’s default for distinguishing type III effectors is at RI=50. RIs for PSI-BLAST predictions are normalized to fall in the range [50,100] to correspond to the range of *de novo* predictions.

S3 Table: Predictions of type III secretion system in 862 bacterial and 90 archaeal proteomes

The table is available as a separate file in excel format.

***Data:*** pEffect predictions of type III effector proteins in completely sequenced 862 bacterial (of which 588 are gram-negative and 274 are gram-positive bacteria) and 90 archaeal proteomes downloaded from the European Bioinformatics Institute (EBI: <http://www.ebi.ac.uk/genomes/>). Column names are as follows:

*TaxID:* taxonomic identifier of an organism

*Org_Name:* name of an organism

*Type:* whether organism is a bacteria or archaea

*Gram:* gram staining, applies only to bacteria

*Size:* size of the proteome

*0.001#T3System_element:* number of *T3 Ortholog clusters,* i.e. groups of evolutionary and functionally related type III secretion system proteins (Methods), that are conserved (determined through BLAST[^13^](#_ENREF_13) e-value ≤ 10^-3^) in a particular proteome

*0.001#Outer_membrane_ring:* number of proteins in a proteome with a BLAST hit at e-value≤10^-3^ to any member of the *Outer membrane ring* cluster

*0.001#Inner_membrane_ring:* number of proteins in a proteome with a BLAST hit at e-value≤10^-3^ to any member of the *Inner membrane ring* cluster

*0.001#Cytoplasmic_ring:* number of proteins in a proteome with a BLAST hit at e-value≤10^-3^ to any member of the *Cytoplasmic ring* cluster

*0.001#Export_apparatus:* number of proteins in a proteome with a BLAST hit at e-value≤10^-3^ to any member of the *Export apparatus* cluster

*0.001#ATPase:* number of proteins in a proteome with a BLAST hit at e-value≤10^-3^ to any member of the *ATPase* cluster

*Distance:* evolutionary distance to the root of the phylogenetic tree of 2966 becterial and archaeal taxa, inferred by Lang *et al.*[^17^](#_ENREF_17)

*BLAST:* number of type III effectors predicted by a homology-based transfer of annotation from a PSI-BLAST[^18^](#_ENREF_18) search at e-value < 10^-3^

*SVM:* number of type III effectors with no hits by PSI-BLAST but an annotation through Profile Kernel SVM

*SUM*: total number of predicted type III effectors by pEffect, i.e. use PSI-BLAST prediction if available and profile kernel SVM otherwise *BLAST%:* fraction of type III effectors predicted by PSI-BLAST

*BLAST%:* total number of PSI-BLAST effector predictions divided by the proteome size

*SVM%:* total number of SVM effector predictions divided by the proteome size

*SUM%:* total number of pEffect predictions divided by the proteome size

S4 Table: Top 10 most frequent UniProt keywords of bacterial and archaeal proteins predicted as type III effectors by pEffect

|  | UniProt keywords  *(PSI-BLAST predictions)* | Freq,% | UniProt keywords  *(SVM predictions)* | Freq,% |
| --- | --- | --- | --- | --- |
| *ARCHAEA* | Uncharacterized protein | 30 | Uncharacterized protein | 40 |
|  | Hydrolase | 6 | Oxidoreducatase | 5 |
|  | Cytoplasm | 5 | Plasmid | 5 |
|  | Nucleotide-binding | 5 | Transferase | 4 |
|  | ATP-binding | 5 | Metal-binding | 4 |
|  | Metal-binding | 5 | Flavoprotein | 4 |
|  | Zinc | 4 | FAD | 3 |
|  | Chaperone | 4 | Lyase | 2 |
|  | Coiled coil | 4 | Kinase | 2 |
|  | Plasmid | 3 | Nucleotide-binding | 2 |
|  | UniProt keywords  *(PSI-BLAST predictions)* | Freq, % | UniProt keywords  *(SVM predictions)* | Freq,% |
| *BAC*  *T ER I A*  *(+)* | Uncharacterized protein | 26 | Uncharacterized protein | 26 |
|  | Transferase | 6 | Transferase | 7 |
|  | Hydrolase | 6 | Nucleotide-binding | 6.6 |
|  | Nucleotide-binding | 5 | ATP-binding | 6.5 |
|  | ATP-binding | 5 | Kinase | 3.7 |
|  | Kinase | 5 | Oxidoreductase | 3.7 |
|  | Cytoplasm | 4 | Phosphoprotein | 3.0 |
|  | Serine/threonine-protein kinase | 3 | Metal-binding | 2.3 |
|  | Metal-binding | 3 | Flavoprotein | 2.1 |
|  | Chaperone | 2 | FAD | 2.0 |
|  | UniProt keywords  *(PSI-BLAST predictions)* | Freq,% (in full T3SS) | UniProt keywords  *(SVM predictions)* | Freq,% (in full T3SS) |
| *BAC*  *T ER I A*  *(-)* | Uncharacterized protein | 28 (26) | Uncharacterized protein | 29 (26) |
|  | Hydrolase | 5 (5) | Transferase | 8 (8) |
|  | Cytoplasm | 5 (4) | Nucleotide-binding | 5 (5) |
|  | Transferase | 4 (4) | Kinase | 5 (5) |
|  | Metal-binding | 4 (4) | ATP-binding | 5 (5) |
|  | Nucleotide-binding | 4 (4) | Phosphoprotein | 5 (5) |
|  | ATP-binding | 3 (3) | Oxidoreductase | 2 (2) |
|  | Kinase | 3 (3) | Membrane | 2 (2) |
|  | Chaperone | 3 (2) | Plasmid | 2 (2) |
|  | Zinc | 3 (4) | Transmembrane | 2 (4) |

***Data:*** Top ten most frequent UniProt keywords associated with proteins from 90 archaeal proteomes that are predicted as Type III system (T3S) effectors by pEffect (1,198 proteins are predicted by PSI-BLAST and 3,057 by SVM), 274 Gram-positive bacterial proteomes (10,703 proteins are predicted by PSI-BLAST and 25,263 by SVM), and 588 Gram-negative proteomes (18,939 proteins are predicted by PSI-BLAST and 75,259 by SVM). All numbers are rounded to the nearest digit.

S5 Table: Clusters of T3 Orthologs in 36 bacterial genera as annotated in the T3DB database

| **T3 Ortholog cluster** | **Conserved in all T3DB annotated species?** |
| --- | --- |
| Outer membrane ring | **Yes** |
| Inner membrane ring | **Yes** |
| Cytoplasmic ring | **Yes** |
| Export apparatus | **Yes** |
| Needle assembly | No |
| Needle major subunit | No |
| Needle minor subunit | No |
| Translocon | No |
| ATPase | **Yes** |
| Effector export | No |

T3DB annotates proteins forming the type III secretion system in ten clusters of T3 Orthologs, *i.e.* groups of evolutionary and functionally related type III secretion system proteins. The number of bacterial genera and species considered in T3DB is 36. Proteins in five of ten T3 Ortholog clusters are conserved in all 36 bacterial genera and species in T3DB. T3 Ortholog clusters were downloaded from <http://biocomputer.bio.cuhk.edu.hk/T3DB/T3-ortholog-clusters.php>

S6 Table: Experimental evidence for the type III machinery in 10 randomly chosen completely sequenced gram-negative bacteria

| *Organism name* | *Evidence for the type III machinery?* |
| --- | --- |
| *Burkholderia pseudomallei* K96243 | Yes[^21^](#_ENREF_21) |
| *Chlamydia trachomatis* D/UW-3/CX | Yes[^22^](#_ENREF_22) |
| *Dechloromonas aromatica* RCB | Yes[^23^](#_ENREF_23) |
| *Photorhabdus luminescens* subsp. laumondii TTO1 | Yes[^24^](#_ENREF_24) |
| *Pseudomonas fluorescens* SBW25 | Yes[^25^](#_ENREF_25) |
| *Pseudomonas syringae* pv. tomato str. DC3000 | Yes[^26^](#_ENREF_26) |
| *Waddlia chondrophila* WSU 86-1044 | Yes[^27^](#_ENREF_27) |
| *Azospirillum lipoferum* 4B | Secretion system not studied |
| *Rhodopseudomonas palustris* BisB18 | Secretion system not studied |
| *Treponema denticola* ATCC 35405 | Secretion system not studied |

Ten randomly chosen organisms with a high percentage of by pEffect predicted type III effector proteins (≥5% of the entire proteome) and homologs in all five T3 Ortholog clusters from the T3DB database (Methods).

### S4 Fig:


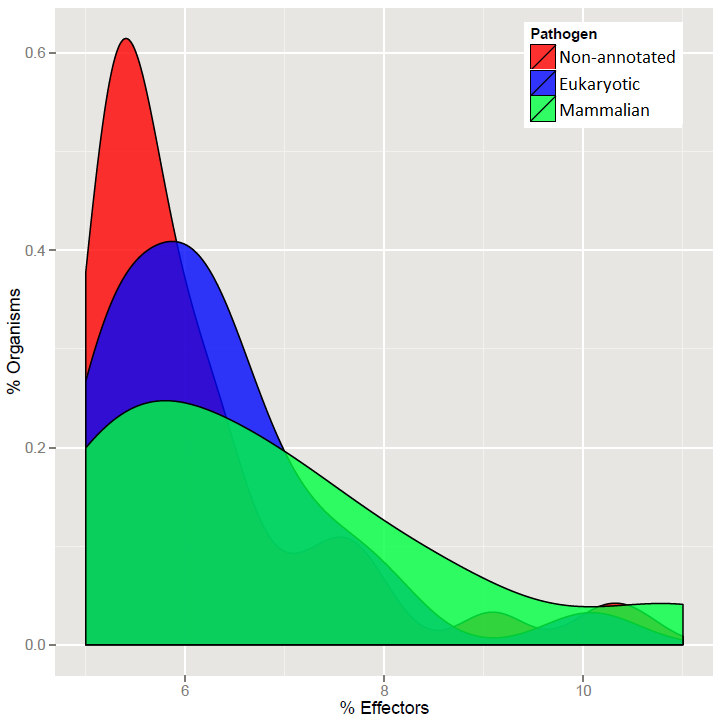


**S4 Fig: Type III effectors most common in mammalian pathogens.** The figure shows the percentage of predicted type III effectors within Gram-negative bacteria in our data set that have more than 5% of the genome dedicated to encoding effectors and a complete T3SS (Methods). Pathogenicity annotations were extracted from the HAMAP database[^28^](#_ENREF_28): (i) red: non-annotated pathogens; (ii) blue: eukaryotic pathogens and symbionts and (iii) green: mammalian pathogens.

*S7 Table: Method performance comparison on a fully independent test set*

|  | *Data set: 10 effector and 390 non-effector proteins* | | |
| --- | --- | --- | --- |
|  | *Acc* | *Cov* | *F_1_* |
| *BPBAac* | 9±21 | 10±21 | 0.1±0.12 |
| *EffectiveT3* | 4±5 | 30±31 | 0.7±0.04 |
| *Modlab* | 3±5 | 20±28 | 0.5±0.04 |
| *T3_MM* | - | - | - |
| *BEAN 2.0* | 3±4 | 30±33 | 0.5±0.04 |
| *De novo* | 50±50* | 20±27 | 0.29±0.24 |
| *PSI-BLAST* | **100*** | 30±32 | 0.46±0.30 |
| *pEffect* | 67±43* | **40±35** | **0.50±0.33** |

Data set: 10 effector proteins added to UniProt after the 2014_02 release and 390 non-effector proteins added to Swiss-Prot after the same release. The data set was sequence homology reduced (at HVAL<0) within itself and with respect to the *Development set* of pEffect (Methods). Thus, this set provides the independent test set for all methods tested.

Methods and performance measures as in Supplementary S1 Table. Highest value in each column is in bold.

Note: T3_MM was not able to produce results for this set during manuscript preparation.

* = unrealistic upper or lower bound given by the standard error due to the small data set size.

# References for Supplementary Information

1 UniProt Consortum. Reorganizing the protein space at the Universal Protein Resource (UniProt). *Nucleic acids research* **40**, D71-75, doi:10.1093/nar/gkr981 (2012).

2 Bairoch, A. & Apweiler, R. The SWISS-PROT protein sequence database and its supplement TrEMBL in 2000. *Nucleic acids research* **28**, 45-48 (2000).

3 Rost, B. Twilight zone of protein sequence alignments. *Protein Eng* **12**, 85-94 (1999).

4 Sander, C. & Schneider, R. Database of homology-derived protein structures and the structural meaning of sequence alignment. *Proteins* **9**, 56-68, doi:10.1002/prot.340090107 (1991).

5 Wang, Y., Huang, H., Sun, M., Zhang, Q. & Guo, D. T3DB: an integrated database for bacterial type III secretion system. *BMC Bioinformatics* **13**, 66, doi:10.1186/1471-2105-13-66 (2012).

6 Efron, B., Halloran, E. & Holmes, S. Bootstrap confidence levels for phylogenetic trees. *Proc Natl Acad Sci U S A* **93**, 13429-13434 (1996).

7 Wang, Y., Zhang, Q., Sun, M. A. & Guo, D. High-accuracy prediction of bacterial type III secreted effectors based on position-specific amino acid composition profiles. *Bioinformatics* **27**, 777-784, doi:10.1093/bioinformatics/btr021 (2011).

8 Cortes, C. & Vapnik, V. Support-vector networks. *Machine learning* **20**, 273-297 (1995).

9 Arnold, R. *et al.* Sequence-based prediction of type III secreted proteins. *PLoS Pathog* **5**, e1000376, doi:10.1371/journal.ppat.1000376 (2009).

10 Lower, M. & Schneider, G. Prediction of type III secretion signals in genomes of gram-negative bacteria. *PloS one* **4**, e5917, doi:10.1371/journal.pone.0005917 (2009).

11 Wang, Y., Sun, M., Bao, H. & White, A. P. T3_MM: A Markov Model Effectively Classifies Bacterial Type III Secretion Signals. *PloS one* **8**, e58173, doi:10.1371/journal.pone.0058173 (2013).

12 Dong, X., Lu, X. & Zhang, Z. BEAN 2.0: an integrated web resource for the identification and functional analysis of type III secreted effectors. *Database : the journal of biological databases and curation* **2015**, bav064, doi:10.1093/database/bav064 (2015).

13 Altschul, S. F., Gish, W., Miller, W., Myers, E. W. & Lipman, D. J. Basic local alignment search tool. *J Mol Biol* **215**, 403-410, doi:10.1016/S0022-2836(05)80360-2 (1990).

14 Finn, R. D. *et al.* The Pfam protein families database: towards a more sustainable future. *Nucleic acids research* **44**, D279-285, doi:10.1093/nar/gkv1344 (2016).

15 Remmert, M., Biegert, A., Hauser, A. & Soding, J. HHblits: lightning-fast iterative protein sequence searching by HMM-HMM alignment. *Nature methods* **9**, 173-175, doi:10.1038/nmeth.1818 (2012).

16 Rice, P., Longden, I. & Bleasby, A. EMBOSS: the European Molecular Biology Open Software Suite. *Trends in genetics : TIG* **16**, 276-277 (2000).

17 Lang, J. M., Darling, A. E. & Eisen, J. A. Phylogeny of bacterial and archaeal genomes using conserved genes: supertrees and supermatrices. *PloS one* **8**, e62510, doi:10.1371/journal.pone.0062510 (2013).

18 Altschul, S. F. *et al.* Gapped BLAST and PSI-BLAST: a new generation of protein database search programs. *Nucleic acids research* **25**, 3389-3402 (1997).

19 Hamp, T., Goldberg, T. & Rost, B. Accelerating the Original Profile Kernel. *PloS one* **8**, e68459, doi:10.1371/journal.pone.0068459 (2013).

20 Kuang, R. *et al.* Profile-based string kernels for remote homology detection and motif extraction. *Proc IEEE Comput Syst Bioinform Conf*, 152-160 (2004).

21 Attree, O. & Attree, I. A second type III secretion system in Burkholderia pseudomallei: who is the real culprit? *Microbiology* **147**, 3197-3199, doi:10.1099/00221287-147-12-3197 (2001).

22 Dai, W. & Li, Z. Conserved type III secretion system exerts important roles in Chlamydia trachomatis. *International journal of clinical and experimental pathology* **7**, 5404-5414 (2014).

23 Salinero, K. K. *et al.* Metabolic analysis of the soil microbe Dechloromonas aromatica str. RCB: indications of a surprisingly complex life-style and cryptic anaerobic pathways for aromatic degradation. *BMC genomics* **10**, 351, doi:10.1186/1471-2164-10-351 (2009).

24 Brugirard-Ricaud, K. *et al.* Variation in the effectors of the type III secretion system among Photorhabdus species as revealed by genomic analysis. *Journal of bacteriology* **186**, 4376-4381, doi:10.1128/JB.186.13.4376-4381.2004 (2004).

25 Mavrodi, D. V. *et al.* Structural and functional analysis of the type III secretion system from Pseudomonas fluorescens Q8r1-96. *Journal of bacteriology* **193**, 177-189, doi:10.1128/JB.00895-10 (2011).

26 Block, A. *et al.* The Pseudomonas syringae type III effector HopG1 targets mitochondria, alters plant development and suppresses plant innate immunity. *Cellular microbiology* **12**, 318-330, doi:10.1111/j.1462-5822.2009.01396.x (2010).

27 Bertelli, C. *et al.* The Waddlia genome: a window into chlamydial biology. *PloS one* **5**, e10890, doi:10.1371/journal.pone.0010890 (2010).

28 Pedruzzi, I. *et al.* HAMAP in 2015: updates to the protein family classification and annotation system. *Nucleic acids research* **43**, D1064-1070, doi:10.1093/nar/gku1002 (2015).
